# Atomic-Level Response of the Domain Walls in Bismuth Ferrite in a Subcoercive-Field Regime

**DOI:** 10.1021/acs.nanolett.2c02857

**Published:** 2022-12-02

**Authors:** Oana Condurache, Goran Dražić, Tadej Rojac, Hana Uršič, Brahim Dkhil, Andraž Bradeško, Dragan Damjanovic, Andreja Benčan

**Affiliations:** † Electronic Ceramics Department, Jožef Stefan Institute, 1000 Ljubljana, Slovenia; ‡ Jožef Stefan International Postgraduate School, 1000 Ljubljana, Slovenia; # National Institute of Chemistry, 1001 Ljubljana, Slovenia; § CentraleSupélec, Laboratoire Structures, Propriétés et Modélisation des Solides, Université Paris-Saclay, 91190 Gif-sur-Yvette, France; ∥ Institute of Materials, Swiss Federal Institute of Technology−EPFL, 1015 Lausanne, Switzerland

**Keywords:** Domain Walls, in situ STEM, Ferroelectric Switching, Bismuth Ferrite

## Abstract

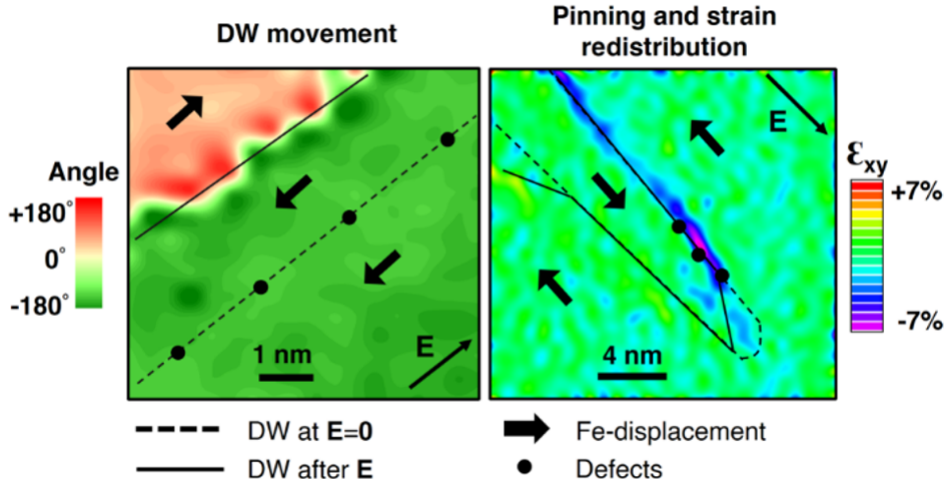

The atomic-level response of zigzag ferroelectric domain walls (DWs) was investigated with in situ bias scanning transmission electron microscopy (STEM) in a subcoercive-field regime. Atomic-level movement of a single DW was observed. Unexpectedly, the change in the position of the DW, determined from the atomic displacement, did not follow the position of the strain field when the electric field was applied. This can be explained as low mobility defect segregation at the initial DW position, such as ordered clusters of oxygen vacancies. Further, the triangular apex of the zigzag wall is pinned, but it changes its shape and becomes asymmetric under electrical stimuli. This phenomenon is accompanied by strain and bound charge redistribution. We report on unique atomic-scale phenomena at the DW level and show that in situ STEM studies with atomic resolution are very relevant as they complement, and sometimes challenge, the knowledge gained from lower resolution studies.

Ferroelectrics, which are characterized by a spontaneous and switchable electric polarization, are currently receiving a great deal of attention from both fundamental and technological points of view as they can be used for memory and logic devices, sensors and actuators, energy harvesters, and photoacoustic modulators, to name just a few examples. When an external electric field is applied to a ferroelectric material, the polarization within the domains tries to align with the direction of the field. As a result, changes occur in the domain structure, causing the domain walls (DWs), which are the boundaries between two adjacent domains with a homogeneous polarization, to move or change their properties. Nanoelectronics aims to exploit the dynamics of DWs, most frequently through their long-range movement during ferroelectric switching.^1^

A new perspective has recently been added to the area of DW nanoelectronics, i.e., the manipulation of the properties of stationary DWs under the influence of a subcoercive electric field.^2^ For instance, in the case of LiNbO_3_, in situ piezoelectric force microscopy has shown how the inclination plane of the DWs and their conductance can be tuned in a relatively weak field regime.^3,4^ The interaction between the DWs and the subcoercive fields was rarely studied,^5−11^ with more data having been collected for high-field ranges.

In situ techniques are essential for a direct observation of the response of DWs to electrical stimuli. In particular, in situ scanning transmission electron microscopy (STEM) provides a high spatial resolution and brings the experiment to the local scale of the DW (i.e., the atomic scale). There are very few in situ STEM studies with atomic resolution targeting ferroelectric DWs^12,13^ compared to in situ STEM studies at the micrometer and nanometer level.^14−17^ The lack of high-resolution in situ studies involving DWs is partly due to the tedious specimen-preparation process and need for special sample holders to apply an electric field. To achieve atomic resolution using the STEM technique, the specimen should be thin enough (ideally below 100 nm) while maintaining the integrity of the electrical device. The need for atomic-resolution studies becomes paramount in the low-field regime because the associated phenomena are expected to be short-range, on the scale of tens of picometers.

In the present study, a bismuth ferrite (BFO) single crystal grown by the flux method (details are given in Supplementary 1, in Supporting Information) used in a capacitor-like configuration was investigated by in situ STEM (Cs-corrected Jeol ARM 200 CF STEM operated at 200 kV combined with a Protochips Aduro system). The goal was to monitor with atomic resolution the DW interaction with a static subcoercive electric field, in a miniaturized capacitor-like device. The hypothesis is that, even in weak (subcoercive) fields, various nano- to atomic-scale phenomena can occur at the wall level, including changes to the wall morphology and in the unit-cell distortion.

We employed a focused ion beam (FIB) (Helios Nanolab 650 with Ga ions source) to prepare the specimen on electrical biasing optimized chips.^18^ The specimen was prepared as with the classic lift-out lamella method and fixed between two electrodes by Pt deposition (more details in Supplementary 2, in Supporting Information). We assume that the external electric field in the analyzed area is relatively homogeneous as we determined from finite element (FE) simulations (Supplementary 2, in Supporting Information).

Bright-field (BF) and high-angle annular dark-field (HAADF) images were acquired simultaneously and represent the same area, at the same scale. The specimen was oriented along the [100]_pc_ (for indexing, pseudocubic pc notation was used). As HAADF imaging provides almost pure atomic-number contrast and is, thus, more robust and less sensitive to mistilts than BF,^19^ the atomic coordinates and displacements, visualized by Fe-displacement vector maps, are determined from HAADF images throughout the publication (more details in Supplementary 3, in Supporting Information). The Fe-displacement vector is qualitatively related to the projected polarization; the displacement is proportional to, but points in the opposite direction to, the polarization.^19^

As shown by BF-STEM (Figure 1a), the DW region appears as a region of lower intensity (darker contrast) due to the presence of strain fields. The domain structure of a BFO single crystal exhibits a high degree of order and consists of zigzag domains embedded in lamellar features. This domain structure appears to be characteristic of a BFO single crystal grown using the flux method.^20,21^ Calculations^22^ attribute their formation and stabilization to competition between long- and short-range electrostatic forces. The same domain configuration was reported in TEM specimens that were not confined to a capacitor-like geometry^20,21^ and in bulk polished samples for piezoresponse force microscopy.^20^

**Figure 1:**
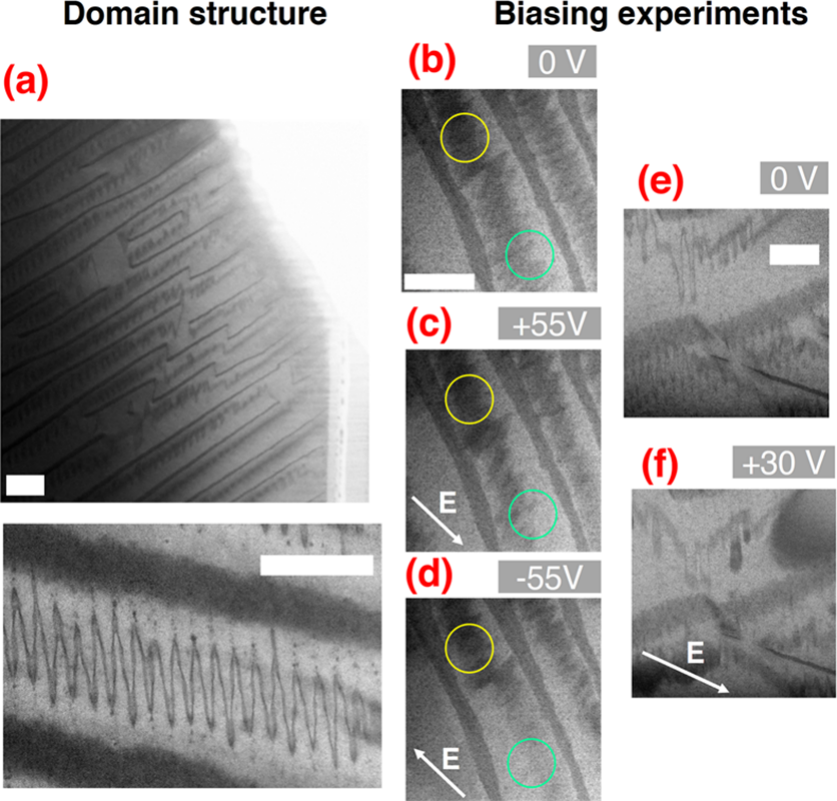
(a) BF images of the domain structure of the BFO single crystal; (b–d) BF images of the electric response of the domain structure at 0 V, +55 V, and −55 V, respectively. Marked with a green circle is a region that appears to respond to the electric field. Regions where the domain structure does not appear to respond to the electric bias are circled in yellow. (e, f) Another example of the evolution of the domain structure with the electric field. At +30 V, the DWs contrast smears off compared to 0 V. The scale bar marks 100 nm.

Jia et al.^21^ performed a comprehensive atomic-resolution investigation of this type of DW in BFO single crystals, employing negative spherical aberration imaging TEM techniques. It was found that the zigzag DWs are ferroelectric 180° walls, while the lamellar ones are ferroelectric–ferroelastic walls. We confirmed the same type of zigzag walls by using HAADF-STEM (more details in Supplementary 3.1 and 3.2, in Supporting Information).

We found that the lamellar domains contain crystallographic defects, most probably related to the crystal-growth process (in Supplementary 3.2, in Supporting Information, antiphase boundaries and dislocations are shown). Nevertheless, the presence of defects on the lamellar DWs was not noted previously.^21^ Irregularities in the TEM atomic contrast have been reported; these were assumed to result from the habit plane of the wall being tilted with respect to the viewing direction. We believe that this does not preclude the presence of defects, which could be concealed if the plane of the wall was not edged-on in the direction of the electron beam.

We applied an electric bias while monitoring the domain contrast (Figure 1b–f). The zigzag DWs are the first to respond to the electric field. At a relatively low magnification, an apparent switching takes place at some sites (circled in green in Figure 1b–d), while other sites do not respond (circled in yellow in Figure 1b–d). DW propagation appears to be favorable to random domain nucleation. Frequently, we cannot switch the zigzag domains, but we observed smearing of the contrast when an electric field is applied (Figure 1e,f), suggesting that a change in the morphology and the structure of the DW takes place. Different regions of the specimen respond differently depending on their own local electric field and strain distribution.^23^ It was previously established that the ferroelectric switching processes are inhomogeneous on local scales.^24−27^

The lamellar features remain fixed in position when the voltage bias is applied, and we associate this with strong pinning on the crystallographic defects.

From the response of the specimen’s domain structure, we conclude that we are working in a so-called low-field regime, i.e., in electric fields below the overall coercive field, where the coercive field is defined as the threshold field to change the orientation of the polarization permanently.^28^ For BFO it is rarely reported in a single crystal but appears to be around a few tens of kV/cm.^29,30^ Comparable values are reported for BFO ceramics,^31^ while more significant values are reported for epitaxial thin films (100–200 kV/cm).^32^ In any case, BFO is a material with a relatively high coercivity for a ferroelectric. For instance, its coercive field is at least 10 times higher than in the prototypical ferroelectric BaTiO_3_.^14^ Moreover, our specimens are thin (less than 100 nm), and it has been reported that the energy to switch the polarization in thin samples increases with decreasing thickness under the same conditions.^13^ Therefore, thin BFO samples lend themselves perfectly to studying the evolution of the DW in subswitching electric field conditions.

We observed the local interactions between the mobile zigzag DWs and the electric field at the atomic level.

During an in situ experiment, we first concentrated on the central region of a segment of the zigzag edge-on DWs (Figure 2a) that is nominally uncharged (Figure 2b).

**Figure 2:**
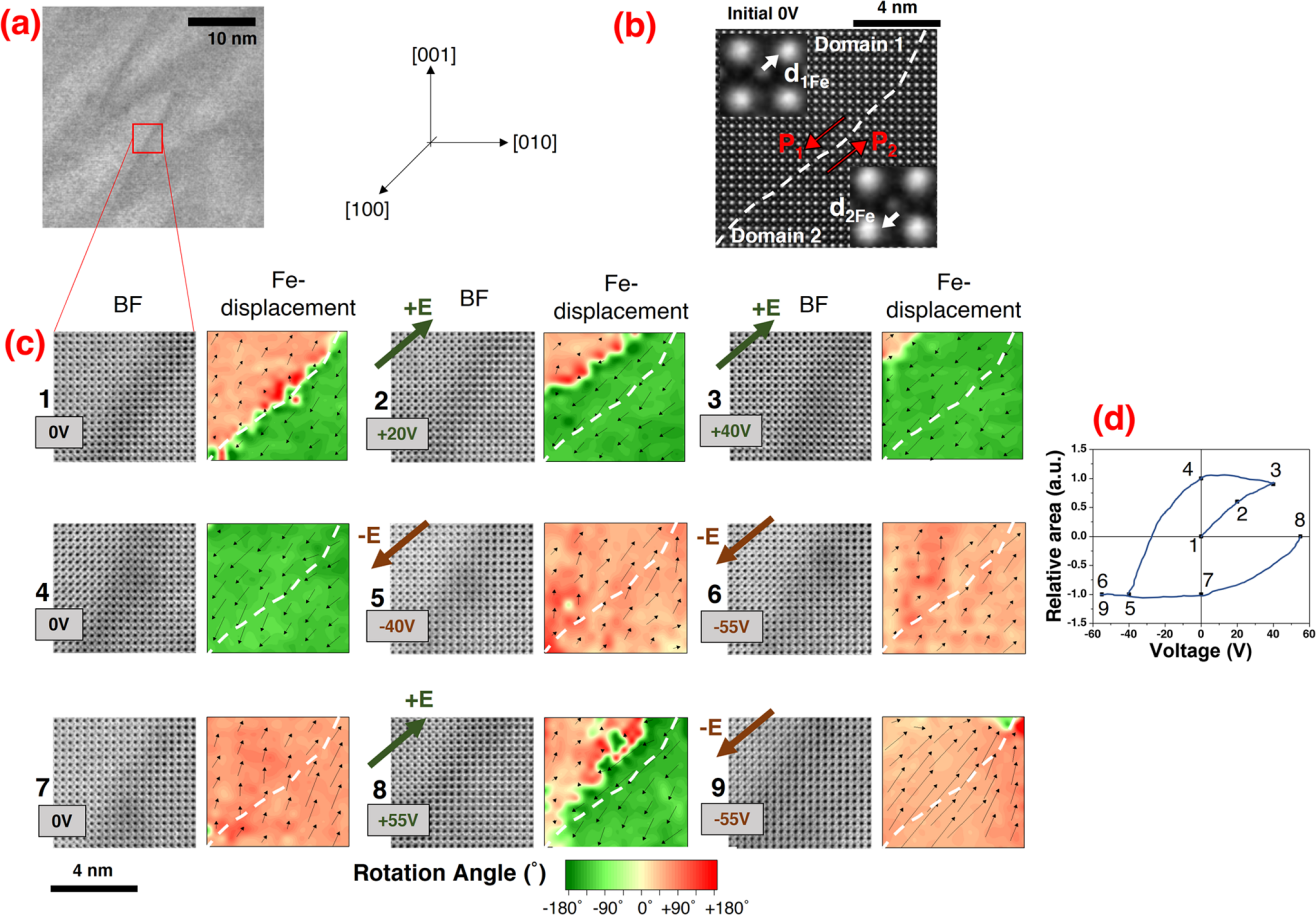
(a) BF image of a zigzag edge-on wall. The site that was further analyzed with atomic resolution is marked with a red rectangle. (b) HAADF image of the DW at the initial 0 V state. The Fe-displacement vector (**d**_**1Fe**_ and **d**_**2Fe**_) and polarization vector (**P**_**1**_ and **P**_**2**_) directions are indicated for each side of the wall. The DW is marked with a white dotted line. The inset is a close-up of one unit cell where the Bi and Fe atomic columns are seen. (c) Atomic-resolution BF-images at the wall location in the [100]_pc_ zone axis together with an Fe-displacement vectors for a few unit cells (black arrows) superimposed on the colored map of the Fe-displacement rotation angle for the sequence 0 V, +20 V, +40 V, 0 V, −40 V, −55 V, 0 V + 55 V and −55 V. The sequence of applied voltages is numbered. The Fe-displacement information was extracted from the HAADF images (see Supplementary 4, in Supporting Information). The arrow lengths indicate the relative magnitude of the Fe-site displacement. The direction of the electric field (**E**) is indicated. A white dotted line marks the location of the enhanced BF contrast. (d) Plot of the relative area difference between Domain 2 and Domain 1 as a function of the applied voltage.

The contrast at the original position of the DW in the BF images during biasing (Figure 2c) is slightly altered; however, it does not vanish. Therefore, we took the area with enhanced dark contrast as a reference and kept it in the middle of the analyzed area.

We obtained an apparent switching in the analyzed area when a voltage bias is applied, as the Fe-displacement maps (Figure 2c) show. The projected Fe-displacement follows the direction of the field, i.e., from 0 V to +40 V, the DW seems to move toward the left, and Domain 2 grows. Going with the negative polarity, the polarization switches again, and no apparent DW is present in the analyzed area (Figure 2c, second line: 0 V, −40 V, and −55 V). When the field is applied directly from 0 V to +55 V, we can bring the DW back in the viewing area. The plot of the area difference between Domain 2 and Domain 1, relative to the total analyzed area, as a function of the voltage is shown in Figure 2d. The plot resembles a macroscopic polarization vs field hysteresis loop and is slightly asymmetric with respect to the voltage axis, an asymmetry that is characteristic for local measurements.^27,33^ As far as we know, this is a rare case where a one-domain-wall hysteresis is derived from the displacement of individual atomic columns.

It is clear that by applying a voltage bias we can move the initial ferroelectric DW by a few nanometers relative to the position of the enhanced BF contrast. This result is intriguing because BF contrast is often considered the landmark of domain structure when describing its evolution with the electrical stimuli in low-magnification STEM in situ experiments.

Here we show that BF-enhanced contrast and the DW do not coincide, and they respond independently when a voltage bias is applied. We propose a scenario to explain this matter which assumes that point-charged defects are accountable for the strain contrast (BF contrast), marking the presence of the DW at 0 V. When a bias voltage is applied, the DW moves, but the defects do not follow the wall movement. Considering that the strain field contrast is smearing, we can hypothesize that the defects migrate and redistribute for a few unit cells. Therefore, the defects that are present must have low diffusivity. A similar scenario was proposed by Stolichnov et al.^34^ Among the possible point-charge defects in BFO systems, Bi-vacancies have been reported together with Fe^4+^, the latest being linked with enhanced electrical conductivity at the wall.^31^ In our crystals, electron-energy-loss spectroscopy (EELS) did not show the presence of Fe^4+^ (Supplementary 6.3, in Supporting Information), and this was also supported by conductive atomic force microscopy, which showed no enhanced DW conduction in the pristine BFO single crystal (Supplementary 6.4, in Supporting Information). Despite no observable drop of the integrated Bi atomic columns’ intensity at the DW position in the HAADF images (Supplementary 6.1, in Supporting Information), we cannot rule out the segregation of the Bi-vacancies at original the DWs, as a large number of Bi-vacancies (up to 30 at. %)^31^ should be present for them to be detected. Our EELS analysis indicated that O-vacancies are present at the zigzag walls with a smaller intensity of the O K edge at the wall location compared to the domain matrix (Supplementary 6.5, in Supporting Information). However, in the present study, the unequivocal detection or quantification of O-vacancies is beyond the capabilities of the method. While O-vacancies are generally considered highly mobile, their mobility can be reduced by ordering at high densities^34^ or by forming defect complexes with Bi-vacancies.^35^

The second experiment tracked the interaction between the charged apex (i.e., the needle-domain’s extremity) of the zigzag walls and the electric bias voltage at the atomic level (Figure 3a). The apex is strongly tail-to-tail negatively charged (as shown in Figure 3b,c). The intention was to have similar conditions as in the experiment describing the uncharged part of the walls. As the BF images show, the apex of the zigzag DWs changes its morphology under a voltage bias (Figure 3c), i.e., it becomes asymmetric. However, when equal and opposite voltages are applied, the apex plane’s change could not be reversed (the BF image at +30 V is similar to the one at −30 V). The rest of the BF-STEM images for other intermediate voltages are shown in Supplementary 7.1, in Supporting Information. Looking at the Fe-displacement maps in Figure 3c leads to the same conclusions. The plane of the apex becomes asymmetric, and the change is not reversible when the opposite voltage is applied (the rest of the Fe-displacement maps for other intermediate voltages are shown in Supplementary 7). Interestingly, the same asymmetry induced by an electric field in the plane of the tip was reported for needle-like 90° DWs in tetragonal BaTiO_3_^36^ that form at the intersection of three sets of domains.

**Figure 3:**
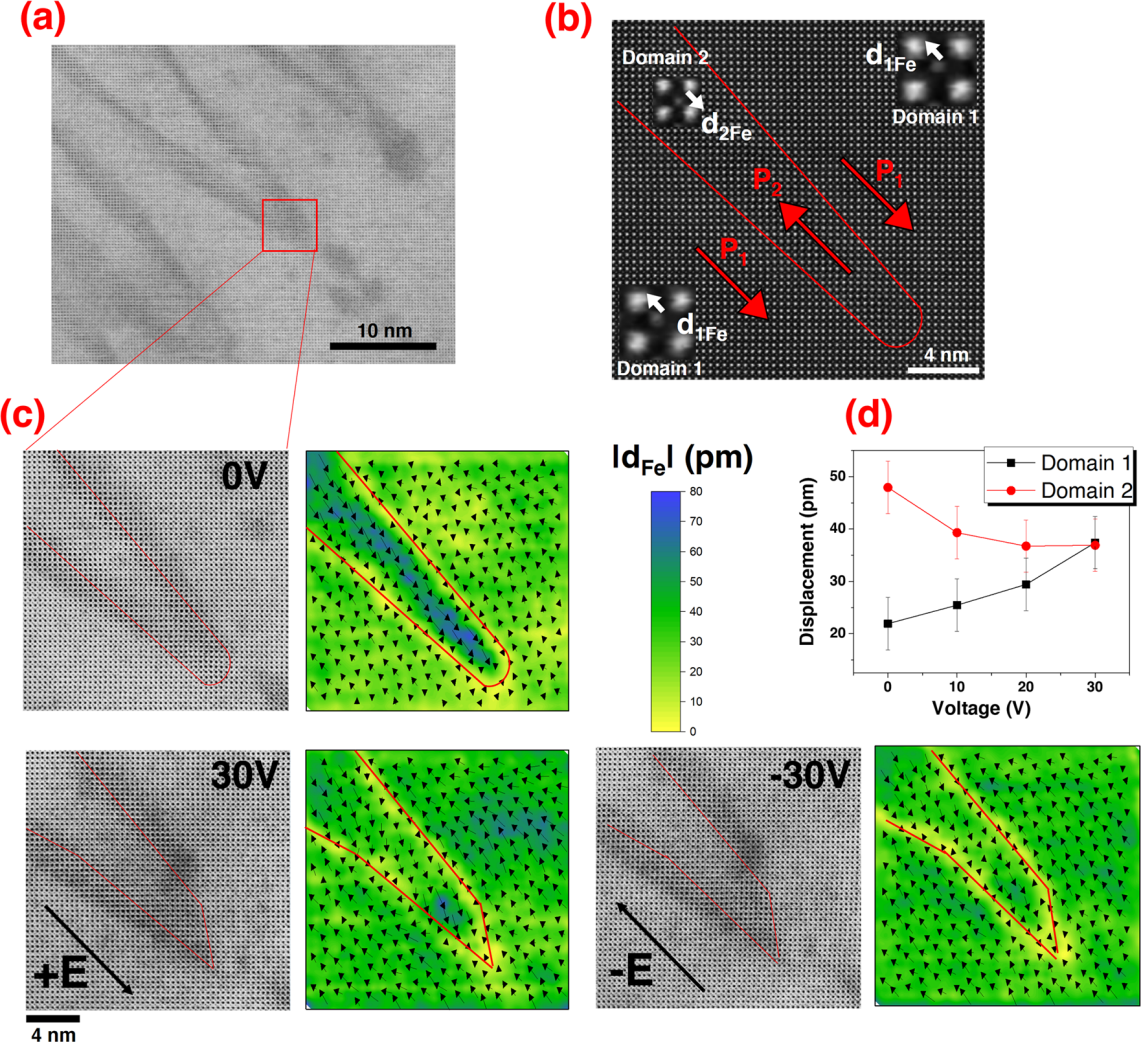
(a) BF image of a zigzag edged-on wall. The site that was further analyzed with atomic resolution is marked with a red rectangle. (b) HAADF image of the DW at the initial 0 V state. The Fe-displacement vector (**d**_**1Fe**_ and **d**_**2Fe**_) and polarization vector (**P**_**1**_ and **P**_**2**_) directions are indicated for each side of the wall. The inset is a close-up of one unit cell where the Bi and Fe atomic columns are visible. (c) Atomic-resolution BF images at the wall location together with Fe-displacement vectors for a few unit cells (black arrows) overlaid on a colored map of the Fe-displacement magnitude for the 0 V, + 30 V, and −30 V sequence. The Fe-displacement information was extracted from HAADF images (see Supplementary 4, in Supporting Information). The arrow lengths indicate the relative magnitude of the Fe-site displacement. The direction of the electric field (**E**) is indicated. The approximative position of the DW is marked with a red line. (d) Fe-displacement vector magnitude as a function of the external applied voltage.

Please note that, for the initial 0 V situation, the magnitude of the projected Fe-displacement is not equal in the two neighboring domains (Figure 3c); a similar structural asymmetry was observed for 90° DWs and is specific to most ferroelectric interfaces.^37^ A noticeable change in the magnitude and orientation of the projected Fe-displacement on either side of the wall occurs when a voltage bias is applied (Figure 3c). The plot in Figure 3d shows how the average (the center of mass) of the Fe-displacement magnitude in the analyzed area changes with the voltage in one domain and the other. The magnitude of **d**_**1Fe**_ in Domain 1 grows (projected polarization **P**_**1**_ in the same direction as the electric field), while the magnitude of **d**_**2Fe**_ in Domain 2 is reduced (projected polarization **P**_**2**_ pointing in the opposite direction than the electric field). The changes in the atomic displacements due to electrical stimuli are almost never discussed in the literature. We show here that, for the weak-field regime in which the experiment takes place, the Fe-displacement, and hence the in-plane polarization, is altered. The Fe displaced about 1.8 times further from the body center at 30 V compared to the initial 0 V configuration in Domain 1, whereas in Domain 2 the Fe-displacement was reduced 1.3-fold. This information might be overlooked in low-magnification in situ studies, where details about the unit-cell distortion are not accessible. A quantitative statement about the polarization change would be inaccurate because we do not have access to the position of the O sublattice. However, qualitatively, we might expect the polarization to follow the same trend as the Fe-displacement. Moreover, the O sublattice displacement is supposed to be higher than the Fe-displacement relative to the body center of the Bi lattice when the electric field is applied.^38^ Therefore, the factors of increasing/decreasing the polarization are expected to be higher than those for the Fe-displacements.

Because of the interesting charge state of the zigzag DWs, a question arises about the defect compensation in the vicinity of the strongly charged tip.^39^ As discussed above, we did not detect any Fe^4+^ in this work. Segregation of Bi-vacancies is suggested by the intensity drop of the atomic columns of Bi in the vicinity of the tip (Figure 4a) (the Bi intensity maps for all voltage ranges are shown in Supplementary 6.2, in Supporting Information). This analysis suggests a higher segregation of Bi-vacancies at the apex compared to the midsection of the zigzag walls, which could be a reason for the observed pinning. If we assume that the negatively charged tail-to-tail apex could attract defects with a net positive charge, we can expect the presence of O-vacancies forming complexes with Bi-vacancies.^35^

**Figure 4:**
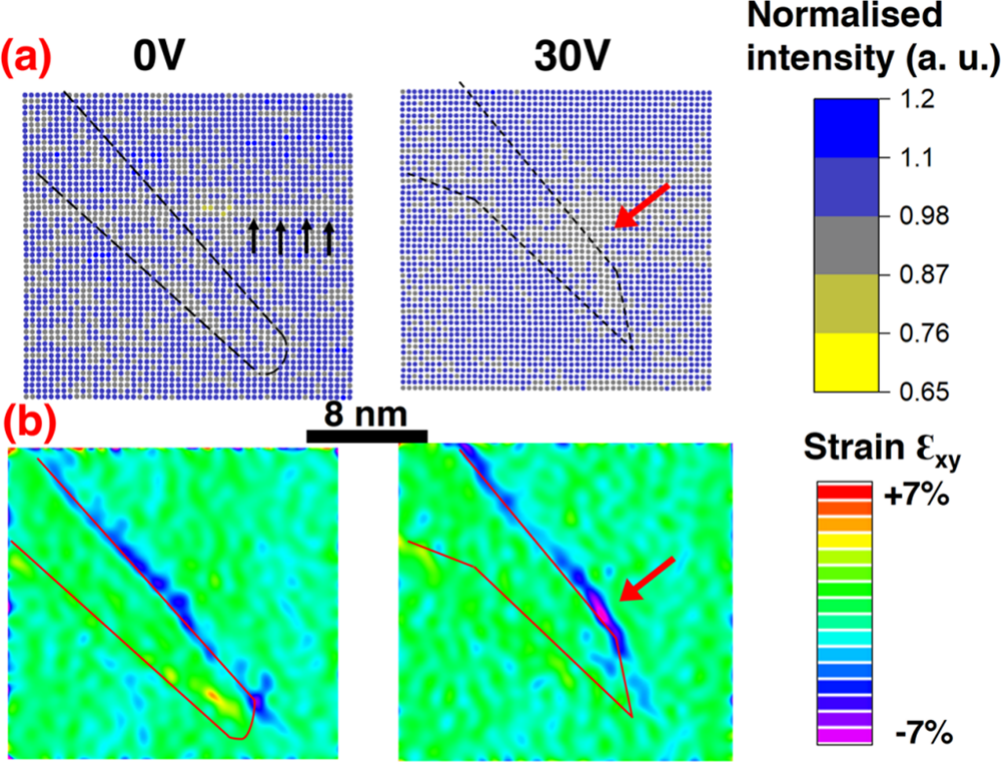
(a) Normalized Bi atomic columns’ intensity maps for 0 and 30 V. The black line marks the position of the DW. Note that the intensity drop extended along the horizontal is likely due to a scanning artifact and it is marked with black arrows. (b) Strain maps calculated by Geometric Phase Analysis for 0 and 30 V. The DW region is marked with a red line. For 30 V in panels a and b, the red arrow marks the location where the intensity drop is substantial and the strain is enhanced.

The electric field most likely influences the redistribution of the Bi intensities, concomitantly with a redistribution of the strain evaluated by the geometric phase analysis (Figure 4). At 30 V there is an apparent segregation of the Bi-vacancies where the abrupt change in the apex plane takes place (Figure 4a), which coincides with an increase in the local strain (Figure 4b). Moreover, we reveal in Supplementary 8, in Supporting Information, that under the influence of the electric field, the polarization bound charges redistribute in the vicinity of the walls as well (as a result of the change in the plane of the wall and the change in the Fe-displacement magnitude and orientation). Our simplified considerations show that at 30 V the bound charges increase on the segments (DWI, DWII) but decrease at the apex compared to the initial 0 V configuration.

Different charge states across the zigzag wall (uncharged midsection vs tail-to-tail charged apex) might set expectations of different dynamics when a voltage bias is applied. In the case of needle-like non-180° ferroelectric/ferroelastic DWs, it is assumed that the apex site will be activated first when the electric field is applied.^13,15,40^ In the present experiment, however, the negatively charged apex seemed to be pinned, while a short-range switching took place for the uncharged central part of the zigzag walls. A higher concentration of defects (O-vacancies + Bi-vacancies) segregating at the apex could explain this experimental observation.

In summary, we applied a dedicated capacitor-like in situ STEM technique to observe the interaction between the weak subcoercive electric field and the zigzag 180° DWs in a BFO single crystal with atomic resolution. We monitored short-range movement with respect to the viewing plane for the neutral segment of the zigzag walls. The DW movement determined from the Fe-displacement map is decoupled from the strain field marked by an enhanced contrast in BF images. This can be explained as low mobility defect segregation at the initial DW position, such as ordered clusters of O-vacancies. In contrast, the zigzag wall’s apex is pinned but atomic displacement response is observed when the electric field is applied. The pinning of the apex can be related to a higher segregation of Bi- and O-vacancies. Furthermore, we show that we can electrically bend the tip plane and induce different strain and bound charge-state configurations. The induced changes appear to be irreversible when an equal, but opposite, electric field is applied. The zigzag domain morphology is not unique to the BFO single crystal; hence, the current atomistic implications revealed here might be applied to other ferroelectric systems, as well. To the best of our knowledge, this publication represents one of the first experimental works showing in situ the behavior of a ferroelectric material under a subswitching electric field near a DW at the atomic level.

## Supporting Information Available

Additional details including sample preparation and characterization approaches, calculations, images, and displacement maps (PDF)

## Abbreviations

DW: domain wall
STEM: scanning transmission electron microscopy
BF: bight field
HAADF: high-angle annular dark field
