# Supplementary material for: Atomic-Level Response of the Domain Walls in Bismuth Ferrite in a Subcoercive-Field Regime
Source: Nano Lett. 2022 Dec 2;23(2):750–6. doi: 10.1021/acs.nanolett.2c02857 (PMC9881151; doi:10.1021/acs.nanolett.2c02857)
Supplement: Supplementary file 1 — nl2c02857_si_001.pdf [file nl2c02857_si_001.pdf]

Atomic-level response of the Domain Walls in bismuth ferrite in a subcoercive-field regime

Oana Condurache †, ‡, *Goran Dražić* †, ‡,#, *Tadej Rojac* †, ‡, *Hana Uršič* †, ‡, *Brahim Dkhil* §, *Andraž Bradeško* §, *Dragan Damjanovic* || and *Andreja Benčan* †, ‡

† Electronic Ceramics Department, Jožef Stefan Institute, 1000 Ljubljana, Slovenia; ‡ Jožef Stefan International Postgraduate School, 1000 Ljubljana, Slovenia; # National Institute of Chemistry, 1001 Ljubljana, Slovenia; §CentraleSupélec, Laboratoire Structures, Propriétés et Modélisation des Solides, Université Paris-Saclay, 91190 Gif-sur-Yvette, France; || Institute of Materials, Swiss Federal Institute of Technology–EPFL, 1015 Lausanne, Switzerland.

Key words: Domain Walls, in situ STEM, Ferroelectric Switching, Bismuth Ferrite

Supplementary 1- **Bismuth ferrite (BFO) single crystal preparation and structural characterization**

The single crystals were grown using flux method starting with high purity powders of Fe_2O_3 and Bi_2O_3 with the flux proportion of 0.15/0.85. The mixture was placed into a platinum crucible, and sealed to prevent the loss of bismuth oxide during the thermal treatment. Further, it was heated to 900°C for 4 h and the temperature was decreased slowly (0.5°C/h) till 790°C and then more rapidly

(~100°C/h) to room temperature. Finally, tiny crystals were extracted after washing with boiling dilute (20%) nitric acid.

The purity of the crystal has been confirmed by X-ray diffraction (Figure S1.1) and Raman Spectroscopy (Figure S1.2).

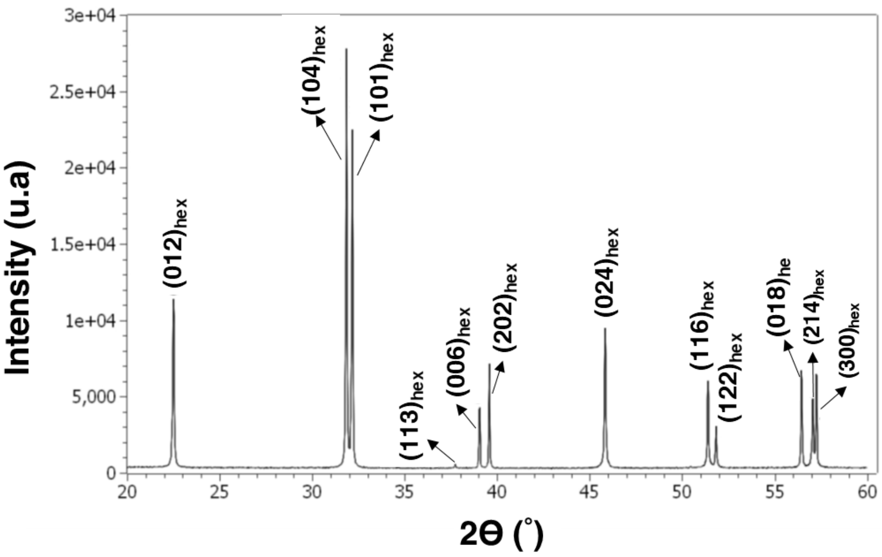

Figure S1.1 X-ray diffraction pattern of crushed crystals. The peaks were indexed according to rhombohedral symmetry with R3c space group. The reported indexation is done in the hexagonal setting. No other peaks other than the ones corresponding to bismuth ferrite (standard card JCPDS no. 86-1518) can be evidenced

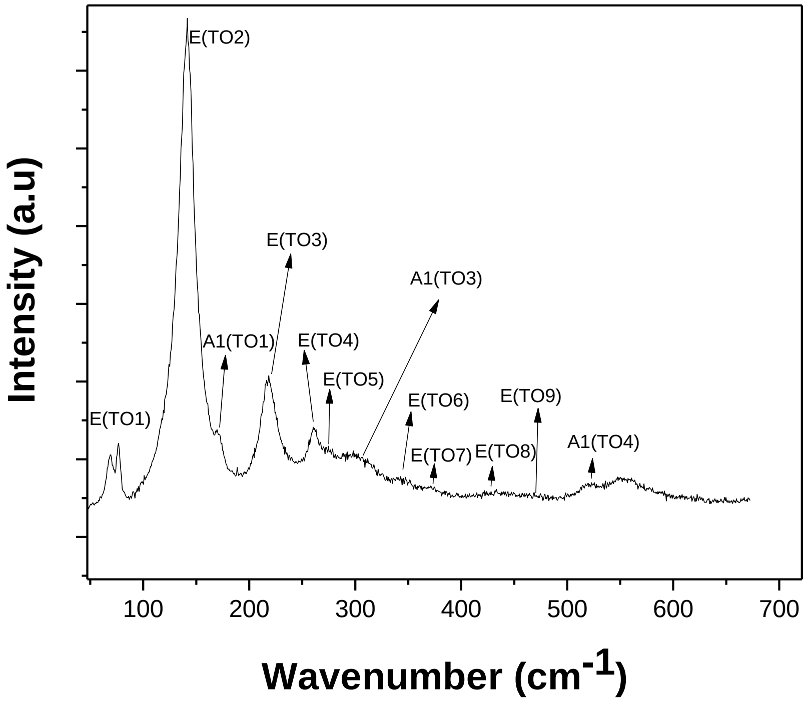

Figure S 1.2 Raman spectrum measured using T6400 Jobin-Yvon, Horiba spectrometer with a green 514nm laser source at room temperature. Only the phonon modes corresponding to bismuth ferrite have been identified. No trace of any parasitic phase can be detected.

Supplementary 2- **Specimen preparation and electrostatic finite element calculations of the electric field distribution in the device**

Specimen preparation

The specimen is prepared on optimized Protochips-Fusion electrical Si-based chips with patterned electrodes by focused ion beam[1] (FIB) (Helios Nanolab 650 with Ga ions source). This type of FIB support chip[1] offers the possibility to prepare sufficiently thin, high-quality specimens in order to achieve atomic resolution while simultaneously applying electric field in situ.

A capacitor-like configuration offers the advantage of a relatively homogeneous electric field [2,3], compared to other more frequently used configurations such as the probe techniques.

The first step of the specimen preparation was electron deposition of a thin layer of Pt (0.4 μm) which allow protection of the top surface of the sample before using the ion beam, which reduces ion implantation and improves sample quality. A next layer of Pt is further deposited with ions to create a thick protection layer for the FIB sample preparation process (2 μm). The sample is then milled using the ion beam to create a standing lamella which is then transferred with the aid of a manipulator on the biasing support chip.

The electrical contacts are made by ion beam assisted Pt-deposition (30 kV, ≈ 0.23 nA). The spacing between the electrodes is by default 20 μm.

A scanning electron microscopy (SEM) image of the specimen on the biasing chip is shown in Figure S2.1a. The specimen is thinned with ions until electron transparency is reached. To maintain mechanical stability, the specimen is not uniformly thinned. Some isolated windows with thickness less than 100nm are done (green color in Figure S2.1a), while the rest of the lamella is kept thicker

≈200 nm (blue color in Figure S2.1a). We always perform the in situ STEM analysis on areas where the domain structure is preserved and which are not thinner than 50 nm. In this way, the effects associated with the reduced thickness (reduction/annihilation of polarization, domain structure alteration) are avoided[4].

Thinning of the specimen was done at 30 kV in 3 steps by progressively lowering the ion beam current (typically 0.8nA, 0.2nA and 8 pA). In addition, low energy ion beam cleaning is performed in order to gradually remove surface amorphization or any other kind of contamination.

The ion beam characteristics that we used for FIB lamella preparation are listed in the table below.

Table S 2 – The ion/electron beam accelerating voltages and currents used during the FIB specimen preparation procedure

Procedure	Voltage	Current
E-beam Pt deposition	2 kV	1.6 nA
I-beam Pt deposition	30 kV	0.2 nA
Electrical contacts	30 kV	0.2 nA
Thinning of the specimen	30 kV	0.8nA
		0.2nA
		80 pA
Cleaning of the specimen	5 kV	40-100 pA
	2 kV	
	1 kV	
	0.5 kV	

Additional cuts are done on top to avoid short circuit through Pt layer when bias voltage is applied. In addition, a cut is made at the bottom of the specimen[2] to remove the material that was redeposited during ion milling and which may potentially be more conductive than the rest.

In order to check the amount of Ga contamination we performed energy dispersive X-ray analysis (EDXS) analysis. Quantitative EDXS/STEM mapping (Bi M, Fe K and Ga L lines shown in Figure S2.2 a)-d)) was done across a BFO lamella prepared by FIB with the same conditions as the samples for in situ studies (a STEM dark field (DF) image is shown in Figure R1 e)). The EDXS-mapping shows an inhomogeneous distribution of Ga throughout the lamella with the highest concentration on the top Pt-deposited layer, as expected. A line EDXS analysis on mid-section (red line marked in Figure S2.2 e)) shows that for the most part the concentration of Ga is around 1 wt% but increases very close to the edge because the specimen is mostly amorphous in this area. The in situ experiments presented in the manuscript are done in regions of the sample which are mostly crystalline. Therefore, we would expect that the Ga concentration was around 1 wt%; we expect that a much smaller amount is incorporated in the perovskite lattice, most of the contamination should be set in the very thin amorphous sidewall.

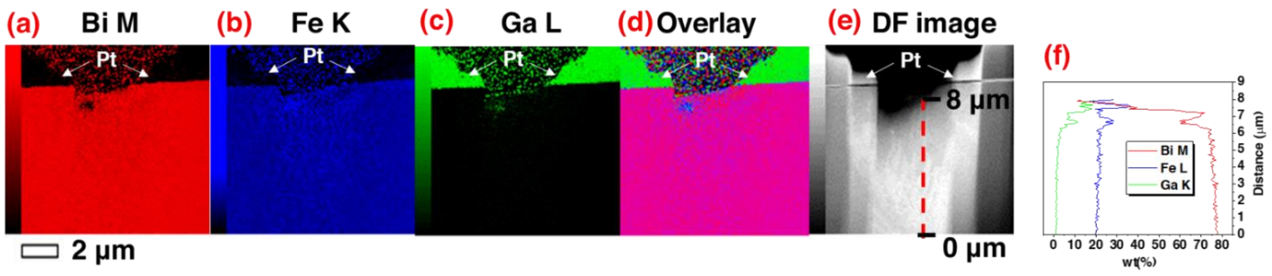

Figure S 2.1 EDXS quantitative elemental maps for (a) Bi M, (b) Fe K, (c) Ga L and their overlaid image (d) for the region of the specimen shown in the DF image in (e). The red dotted line marks the region where the EDXS line analysis show in (f) was taken

Electrostatic finite element model calculations

The electric field distribution was simulated using the finite element method (FEM) implemented in Comsol Multiphysics 5.2 with microelectromechanical systems module. The geometric model (Figure S2a-d) was built considering the three dimensions of the specimen.

The sections used to build the geometric model for FEM are marked with matching colors (Figure S2a, c-d):

- Section 1 (pink): parts which are in direct contact with the electrodes and are relatively thick: 1.2 µm.
- Section 2 (green): electron transparent windows with thickness between 70 and 150 nm.
- Section 3 (blue): electron transparent window of about 200 nm thickness.

Figure S2c-d shows the simplified built geometric model along with in-plane and cross section dimensions used for the electrostatic FEM. The (z,y) dimensions were measured from scanning

SEM) imaging (Figure S2c). The thickness profile (z,x) was determined by scanning transmission electron microscopy (STEM) - electron energy loss spectroscopy (EELS) thickness mapping (Figure S2b) for the very thin sections (2 and 2') and approximated from SEM imaging for the other sections (1, 1', 3) (Figure S2d).

Tetrahedral adaptive mesh was used for the FEM simulation (mesh element maximum 1.6 μm and minimum 0.2 μm). The potential difference between the electrodes was set to +55 V (maximum value applied in the experiment) and it was used as a boundary condition. The relative dielectric permittivity was considered $\varepsilon_{BFO} \approx 80$[5] for the bismuth ferrite and $\varepsilon_{Pt} \approx \infty$ for the Pt layer.

The results of the FEM simulations are shown in Figure S2e-f.

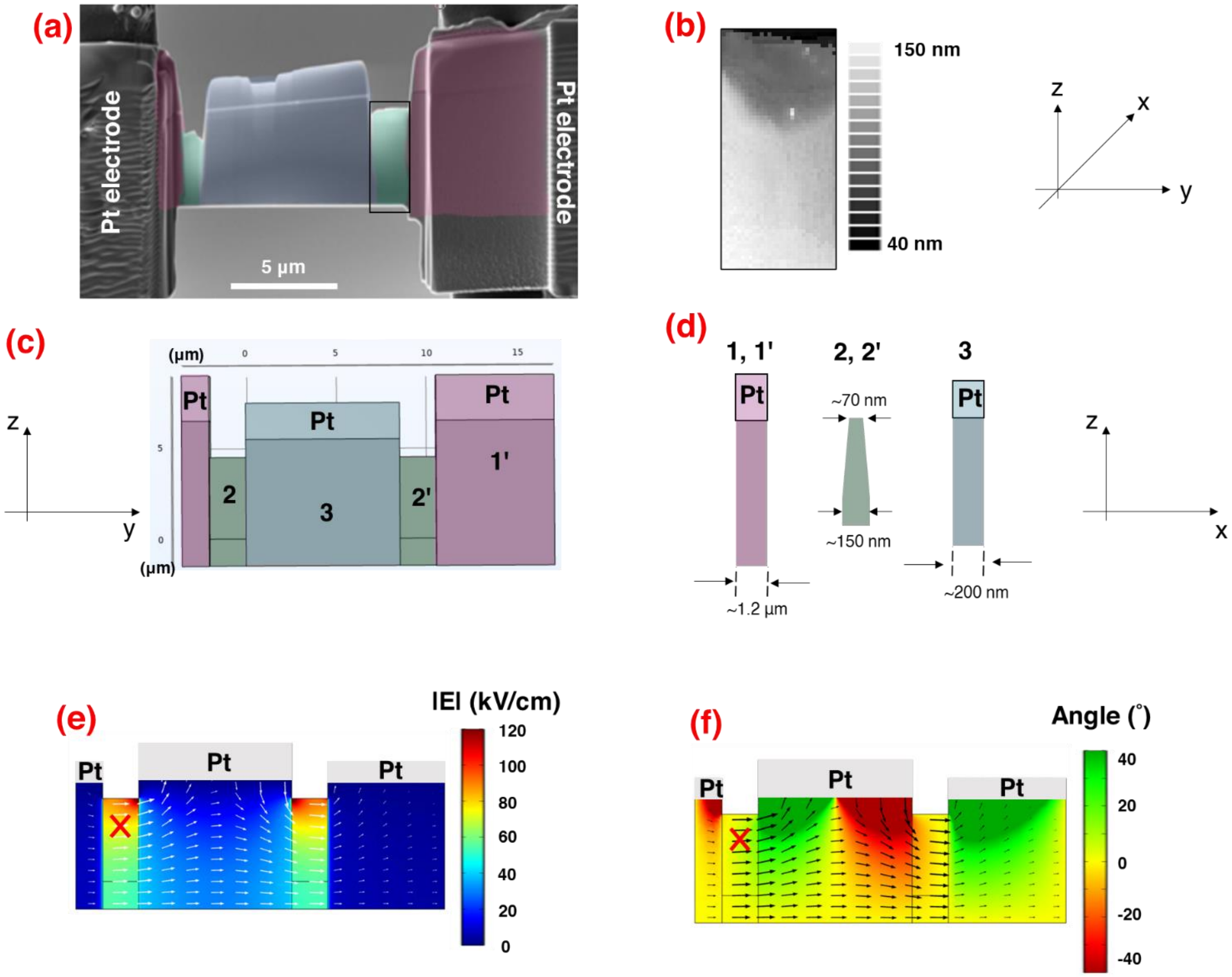

Figure S 2.2 (a) False colored SEM image of the specimen prepared in FIB. The sections of the specimen considered further in the FEM model are colored.

(b) EELS-thickness map of the region marked with black rectangle in (a).

(c) In-plane z-y geometric configuration of the model. (d) The cross-section z-x geometric configuration of the model.

Colored map of the distribution of the electric-field e) magnitude and f) orientation in the specimen as calculated by FEM. White and black arrows, respectively, mark the direction of the field on each location. The density of arrows is arbitrary. With red cross the approximate location of the experiment is indicated.

Supplementary 3- **Types of domain walls in BFO single crystal**

The BFO single crystal presents two types of domain walls morphology as described in the main manuscript: zigzag-like (Figure S3.1) and lamellar-like DWs (Figure S3.2).

From HAADF images acquired in $[100]_{pc}$ zone axis, the coordinates of the atomic columns were determined using a 2D Gaussian fit according to a previously reported methodology[5]. We can identify the position of Fe and Bi. However, the position of O, which accounts for the center of negative charge of the unit cell, is concealed. It has been experimentally demonstrated that the position of O is redundant because Fe-displacement in respect to the Bi sublattice ($\boldsymbol{d_{Fe}}$) gives enough qualitative information on the projected polarization direction: namely, the projected polarization is proportional but points in opposite direction to Fe-displacement vector[6].

In rhombohedral symmetry of BFO (space group $R3c$), the spontaneous polarization can lie along one of the four diagonals in the pseudocubic perovskite unit cell (along the $[111]_{pc}$ direction). If one, two or three components of the polarization vector are reversed in one domain compared to the adjacent domain, the DW will be: 71°, 109° or 180°, respectively[7].

In the case of uncharged configurations (head-to-tail or tail-to-head configuration), in order to minimize the electrostatic and elastic energy, the walls usually lie on (or close to) the neutral plane, namely 71º will lie on {110}pc, 109º on {100}pc and 180º on {110}pc[8-11]. No assumptions can be made about the crystallographic plane in which the charged DWs (head-to-head or tail-to-tail configuration) are lying.

We were able to assign the zigzag walls as being of 180°- type: $\boldsymbol{d_{Fe}}$ is antiparallel in one side compared to the other of the wall and the DW lays approximately on neutral $\{110\}_{pc}$ plane[10] (Figure S3.1).

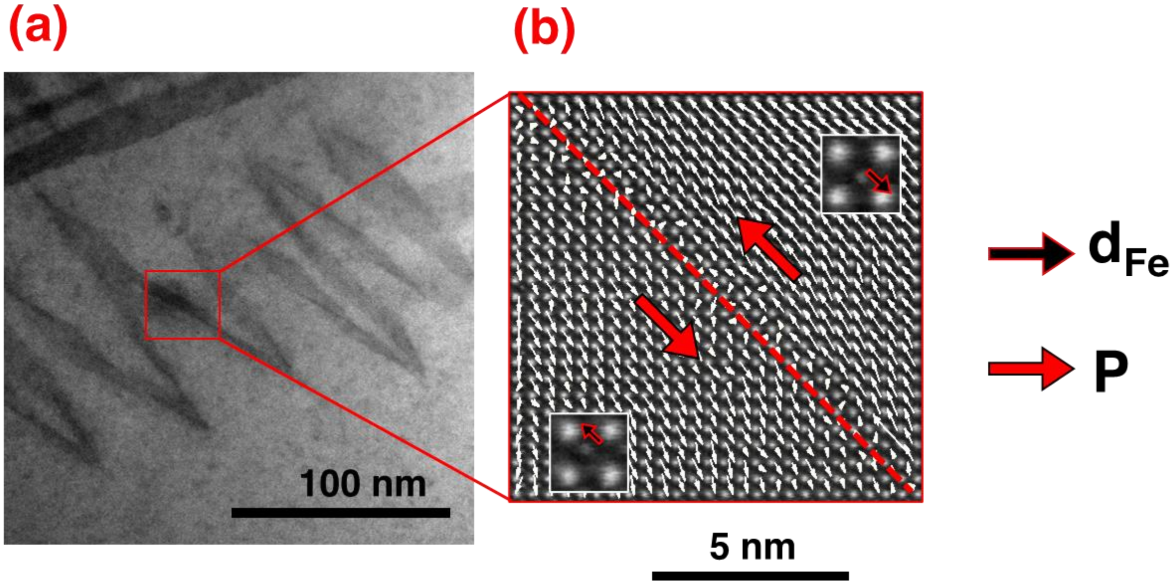

Figure S3.1 (a) Bright field (BF) image of the zigzag domain structure. (b) HAADF atomic resolution image of a region on a zigzag wall with the corresponding Fe-displacement map overlaid. The position of the wall is marked with red dotted line. The insets on each side of the wall show an one-unit cell close-up and the approximate direction of the Fe-displacement (black arrow ($\boldsymbol{d_{Fe}}$)) in respect to the center of Bi lattice. The direction of the polarization is indicated with red arrow ($\boldsymbol{P}$).

The lamellar features appear to contain different crystallographic defects (Figure S3.2a). The crystallographic defects have been identified to be dislocations (Figure S3.2b), and antiphase boundaries (Figure S3.2c-d). Moreover, the lamellar features are nominally head-to-head charged DWs (see the direction of Fe-displacement and projected polarization, respectively in Figure S3.2

c)-d)). Because we have access only to the projected polarization in the imaging plane (we can determine only 2 components out of 3) and the DWs are charged so there are no restrictions on the plane they should lay[10,11], we cannot univocally determine the angle type of the lamellar DWs. They can be either 180˚ or 109˚ DWs. Previous studies[12] report this type of DWs to be ferroelastic so, the 109˚ DWs scenario is more plausible.

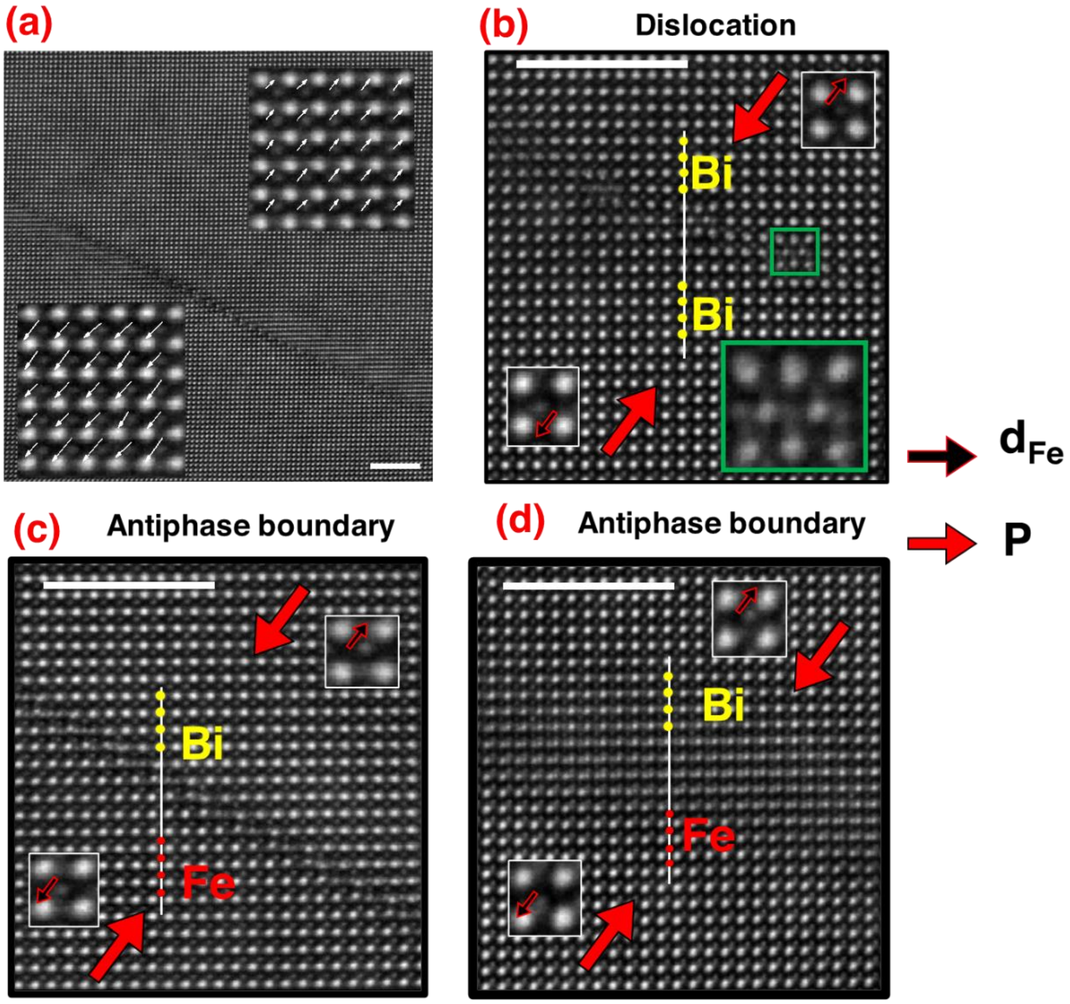

Figure S 3.2 (a) HAADF image of lamellar-like features. On each side of the lamellar-like features a close-up of 5x5 unit cells is shown, together with the overlapped Fe-displacement vectors.

Atomic resolution HAADF image on a location of the lamellar wall showing (b) a dislocation defect (green inset) and (c)-(d) antiphase boundaries (half unit cell shift from one domain to the other is marked: Bi-atomic columns align to Fe-atomic columns).

The white scale bar marks 4 nm. The insets on each side of the defect show an one-unit cell close-up and the approximate direction of the Fe-displacement (black arrow ($\mathbf{d}_{Fe}$)) in respect to the center of Bi lattice. The direction of the polarization is indicated with red arrow ($\boldsymbol{P}$).

Supplementary 4 – **Original HAADF images**

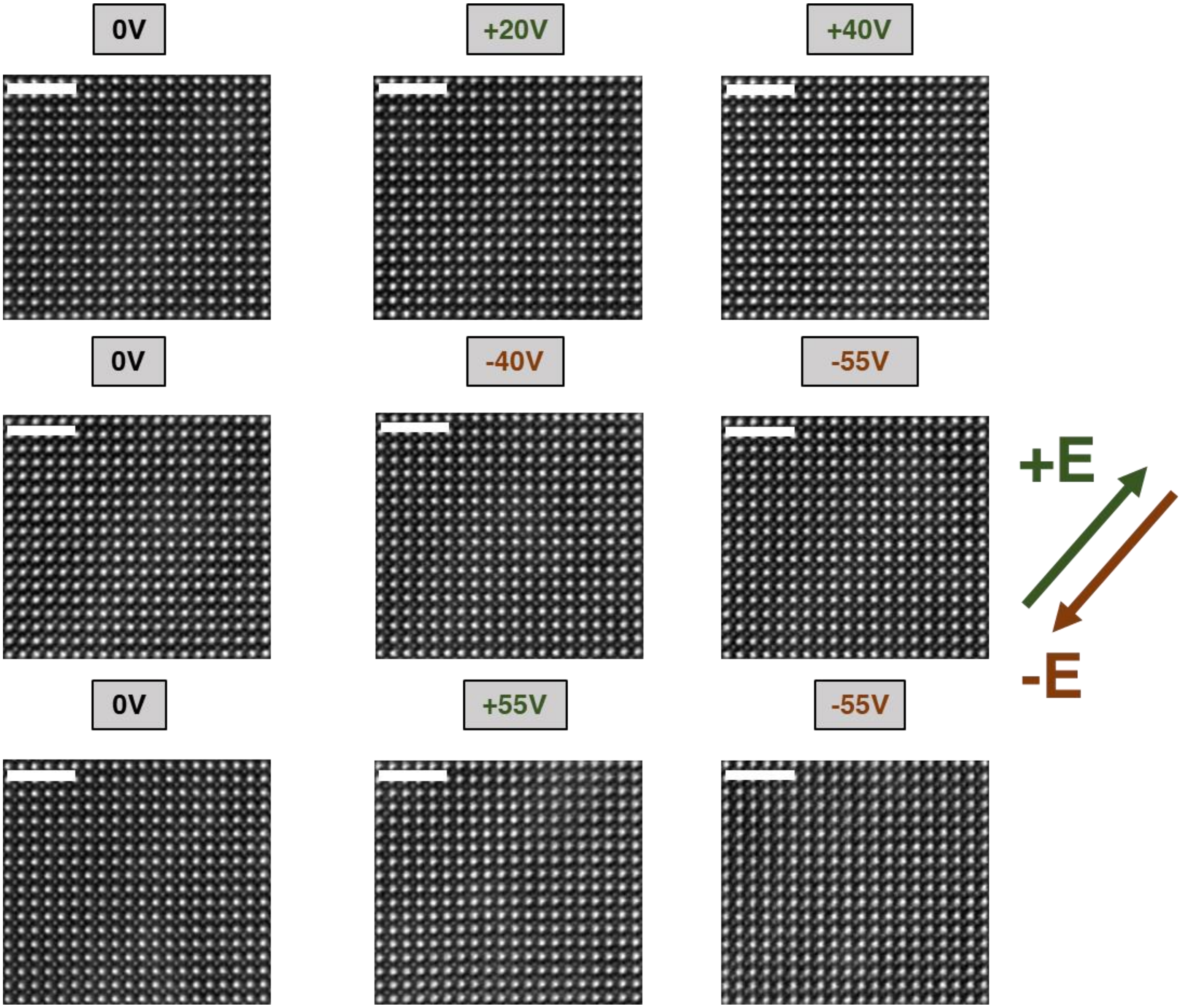

Figure S 4.1 Original HAADF images for the 0V, +20V, +40V, 0V, -40V, -55V, 0V, +55V and -55V sequence. The direction of the electric field is indicated by arrows. The images correspond to the experiment shown in Figure 2 in the main manuscript. Each HAADF image is an integration of 10 frames. The scale bar is 2 nm.

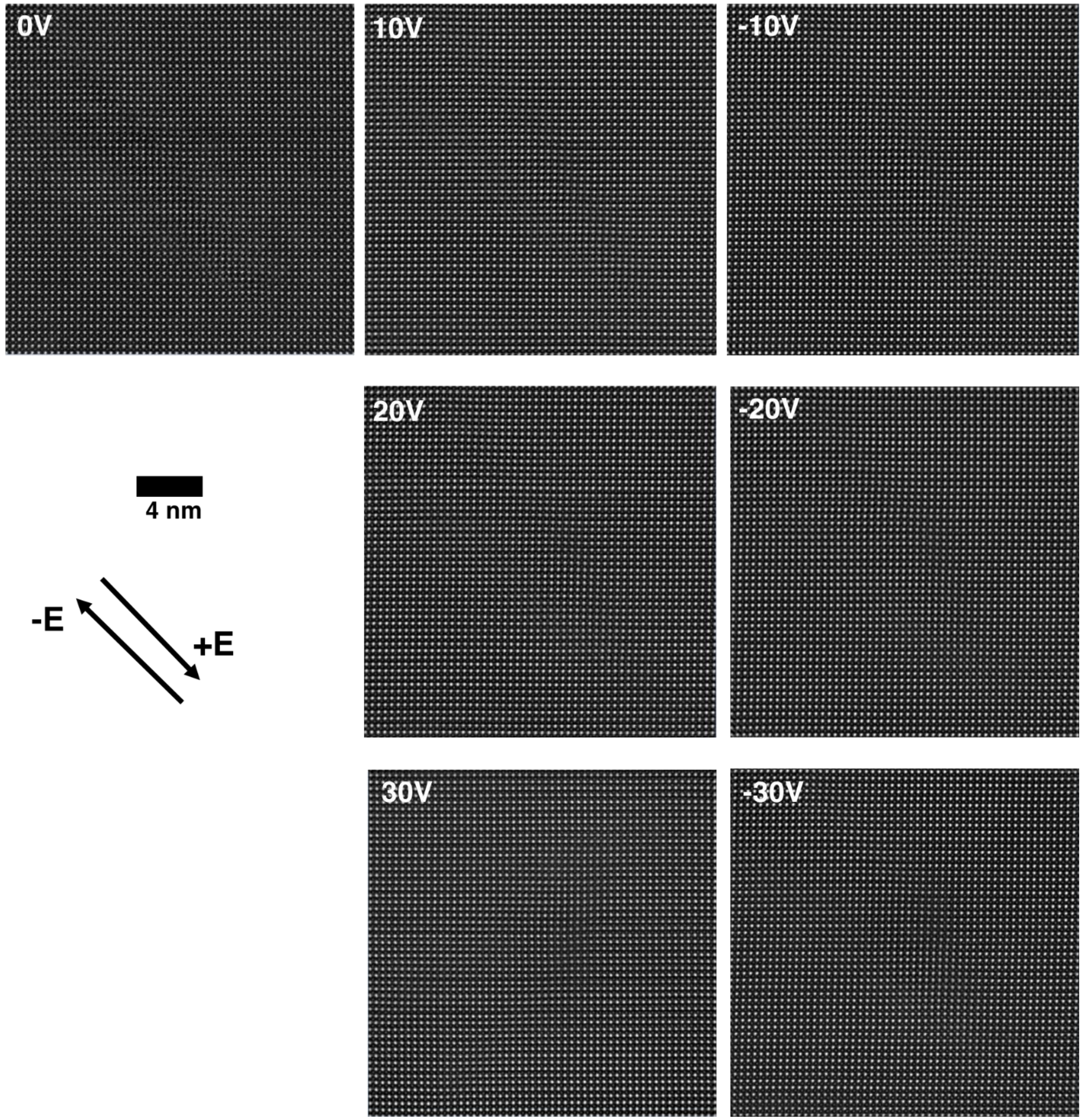

Figure S 4.2 Original HAADF images for the 0V, 10V, -10V, 20V, -20V, +30V and -30V sequence. The direction of the electric field is indicated by arrows. The images correspond to the experiment shown in Figure 3 in the main manuscript. Each HAADF image is one individual frame.

Supplementary 5 - **Displacement maps for all voltages following the experiment shown in Figure 2 in the main manuscript**

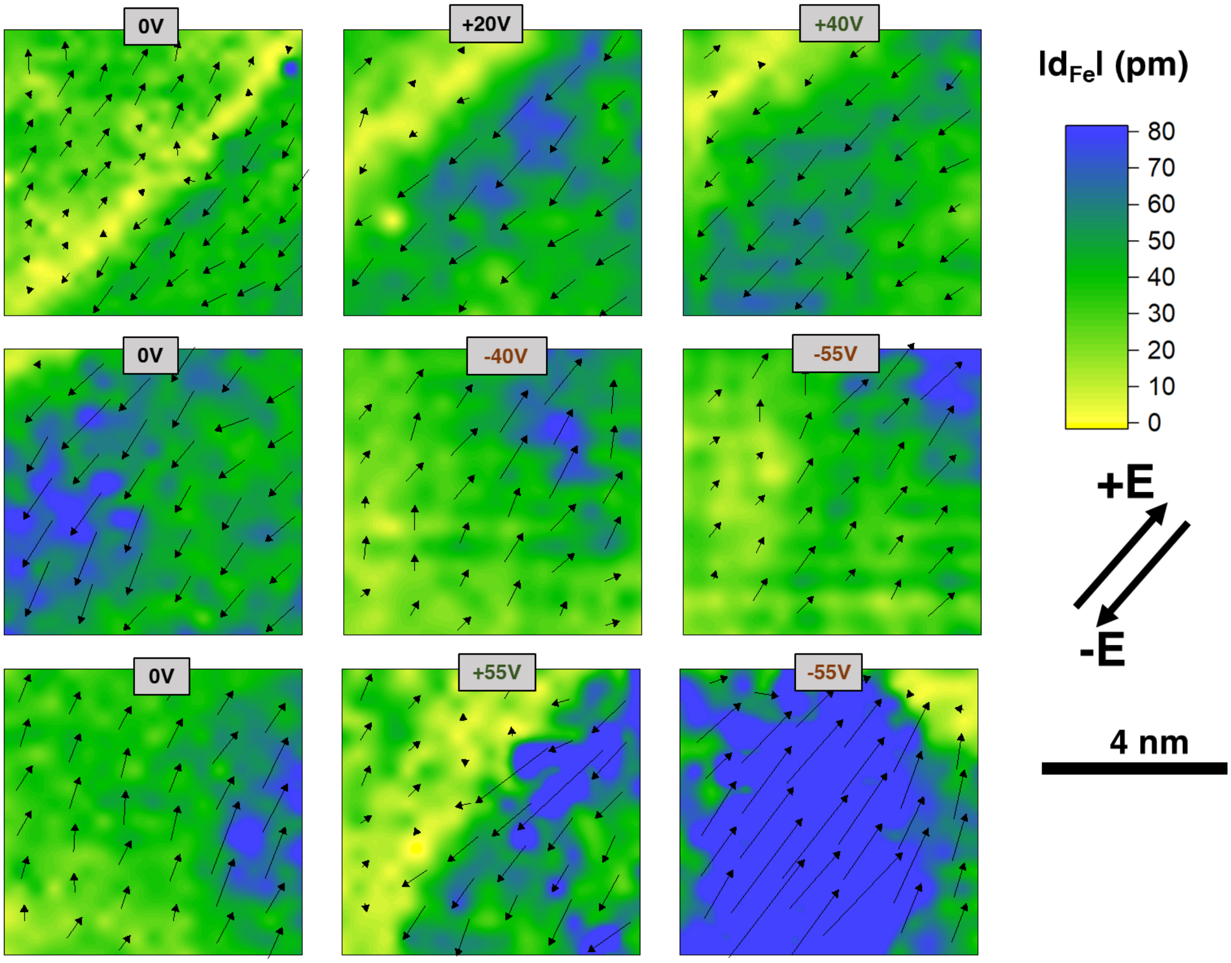

Figure S5 Fe-displacement map (black arrows) superimposed on the colored map of the value of Fe-displacement magnitude for the 0V, +20V, +40V, 0V, -40V, -55V, 0V, +55V and -55V sequence.

Supplementary 6– **Probing possible charged defects on the zigzag DWs.**

Probing Bi vacancies.

The normalized Bi-column intensities were determined from HAADF images using a previously reported method[5,11]. The detector's background intensity is subtracted from the intensity of each pixel in the raw HAADF image. Further, the intensities of the atomic columns were extracted by the integration of the pixel values within one sigma, approximating a Gaussian-type intensity distribution.

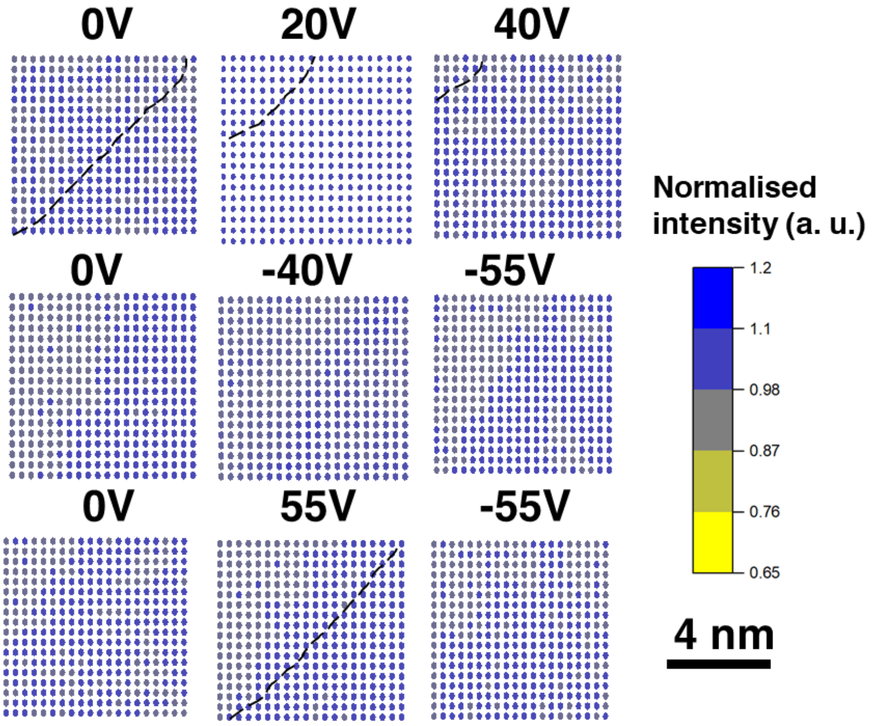

Figure S 6.1 Normalized Bi-atomic columns intensity maps in the following sequence of voltages: 0V, 20V, 40V, 0V, -40V, -55V, 0V, 55V and -55V for the experiment following the central part of the zigzag DWs (Figure 2 in the main manuscript). With black dotted line the position of the DW which moves is marked based on Fe-displacement map (Figure 2 in the main manuscript).

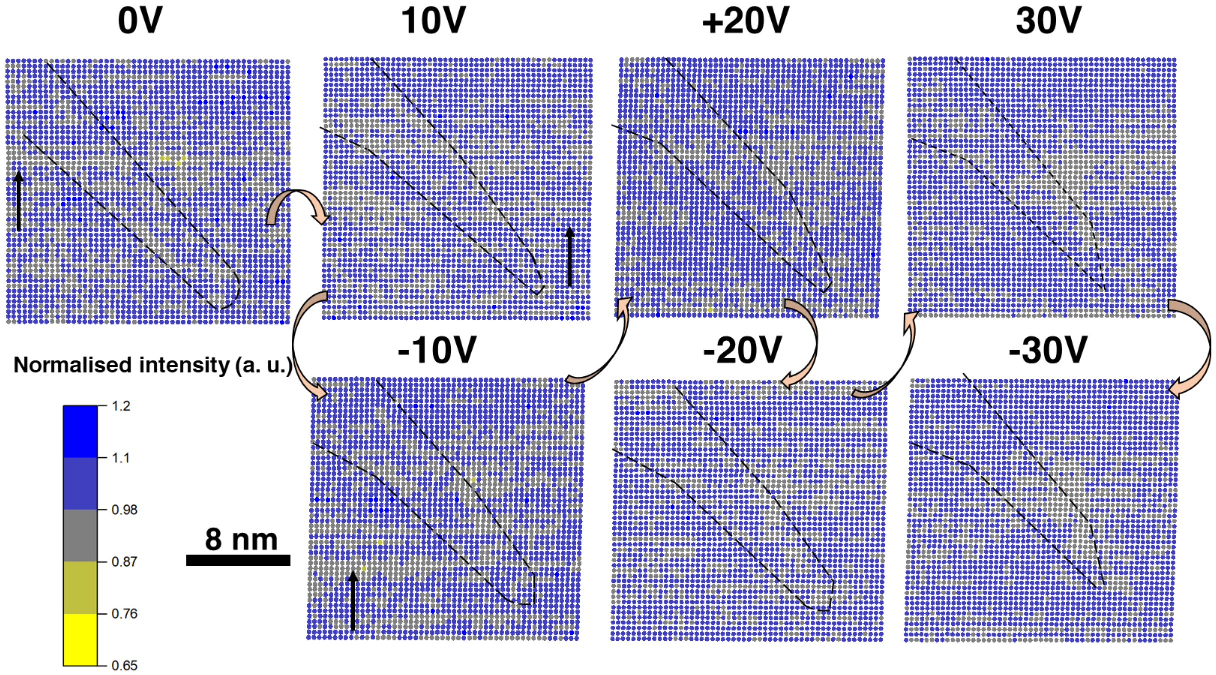

Figure S 6.2 Normalized Bi-atomic columns intensity maps in the following sequence of voltages: 0V, 10V, -10V, 20V, -20V, 30V, -30V for the experiment following the tip of the zigzag DWs (Figure 3 in the main manuscript). With black line the position of the DW is marked based on Fe-displacement map (Figure 3 in the main manuscript). Note that intensity drop extended along the horizontal it is likely due to scanning artefact and it is marked with black arrows

Probing Fe^{4+}

From the EELS spectra we extracted the energy onset difference between O-K and Fe-L_3 edges (chemical shift), which is sensitive to the oxidation state of Fe. Both on and off the DW, we detect a value of the chemical shift of about ΔE=178 eV, fingerprint of Fe^{3+}.[5]

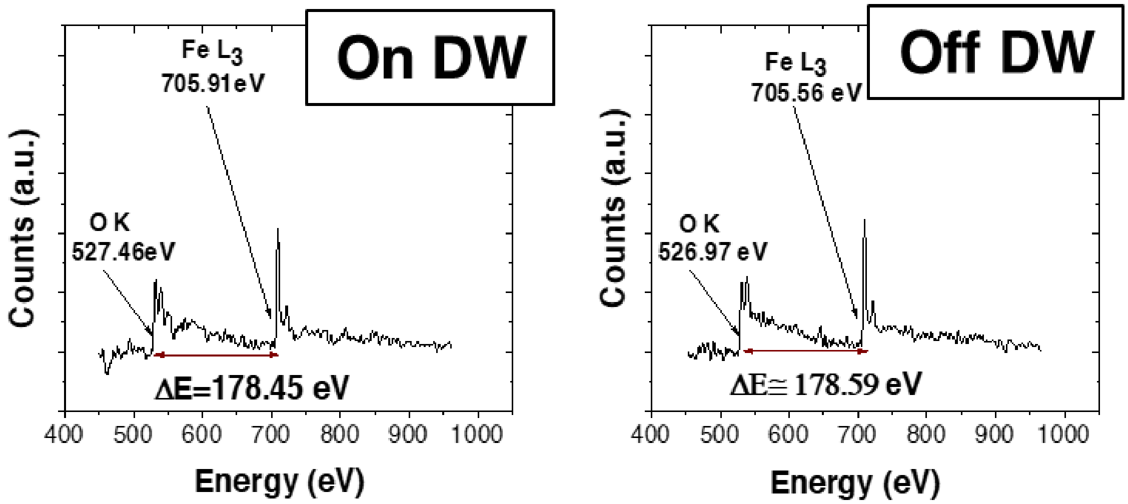

Figure S 6.3 EELS spectra on and off the location of the zigzag DWs. The energy onset ΔE difference between O-K and Fe-L_3 edges is shown.

Probing DWs' conduction

Piezoresponse force microscopy (PFM) and conductive atomic force microscopy (c-AFM) were performed using an atomic force microscope (AFM) Asylum Research, Molecular Force Probe 3D, Santa Barbara, CA, USA. A tetrahedrally shaped silicone AFM tip with a silicone cantilever, both coated with Ti/Ir, was used for the analyses (Asylec, AtomicForce F&E GmbH). Out-of-plane PFM imaging was performed in dual AC resonance tracking mode (DART) using an AC voltage of 10 V. The c-AFM imaging was performed by applying a DC voltage ranging from 5 to 30 V to the tip (the c-AFM image in Fig. S6.4 was acquired at 30V DC). The c-AFM investigation showed no increased conductivity on DWs (Fig. S6.4e).

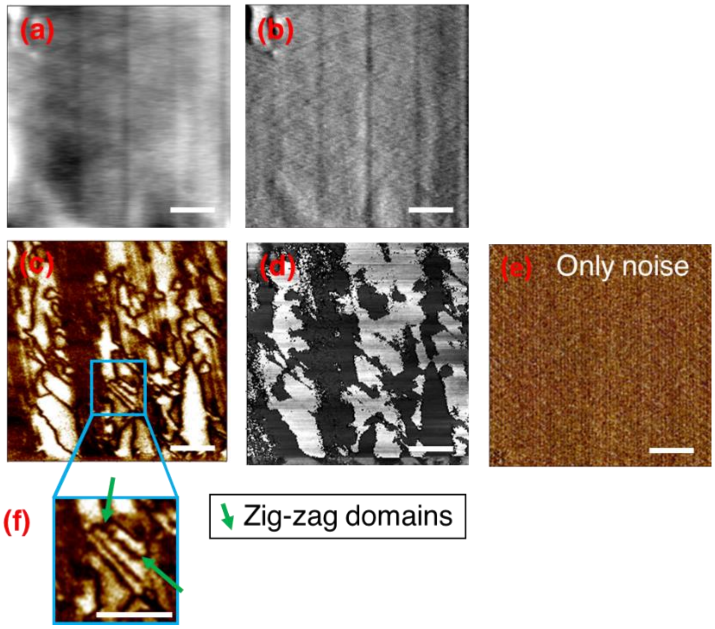

Figure S 6.4 Topography (a) height and (b) deflection; out-of-plane PFM DART (c) amplitude and (d) phase images; (e) c-AFM current image and (f) the closer look to the area marked in panel (c) by blue square. The scale bar marks 1 µm.

O vacancies on zigzag DW

The O K edge intensity appears to be slightly lower in the DW location (marked with black line in Figure S6.5) compared to the domain matrix (marked with red line in Figure S6.5). The present result suggests probable segregation of O vacancies point charged defects at the zigzag walls.

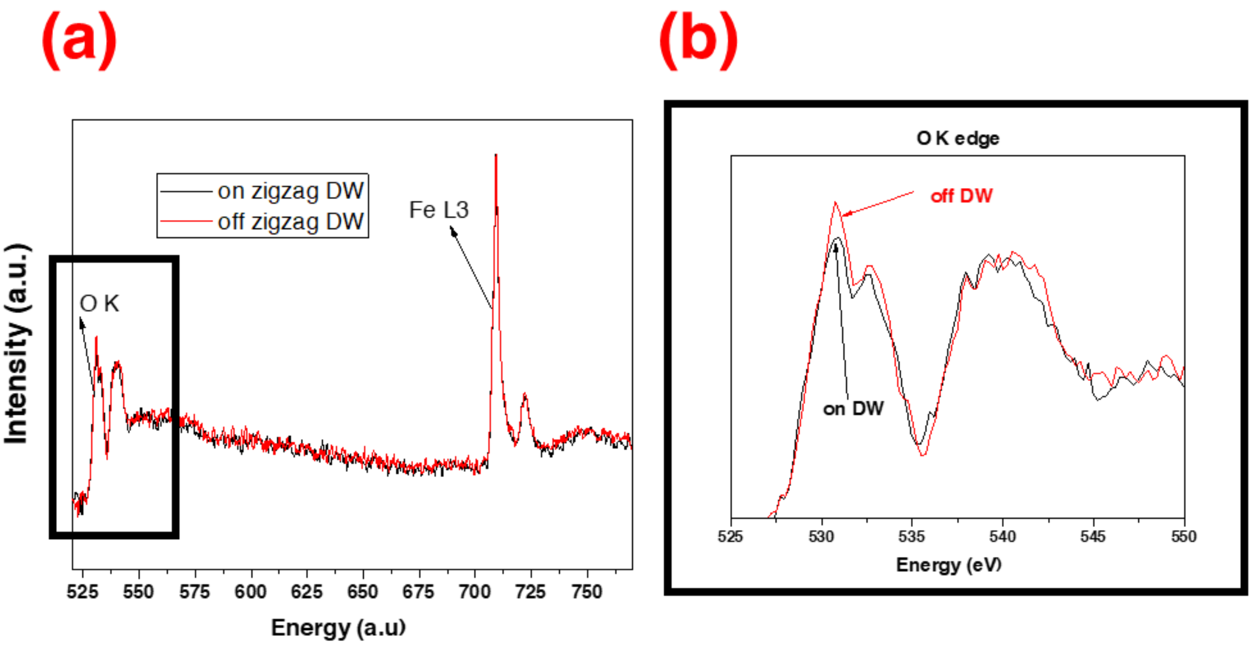

Figure S 6.5 (a) EELS spectra on and off the location of the zigzag DWs. (b) Close-up of the O K edge from the region marked with black rectangle in a).

Supplementary 7 - **BF images and Fe-displacement maps for all voltages following the experiment shown in Figure 3 in the main manuscript**

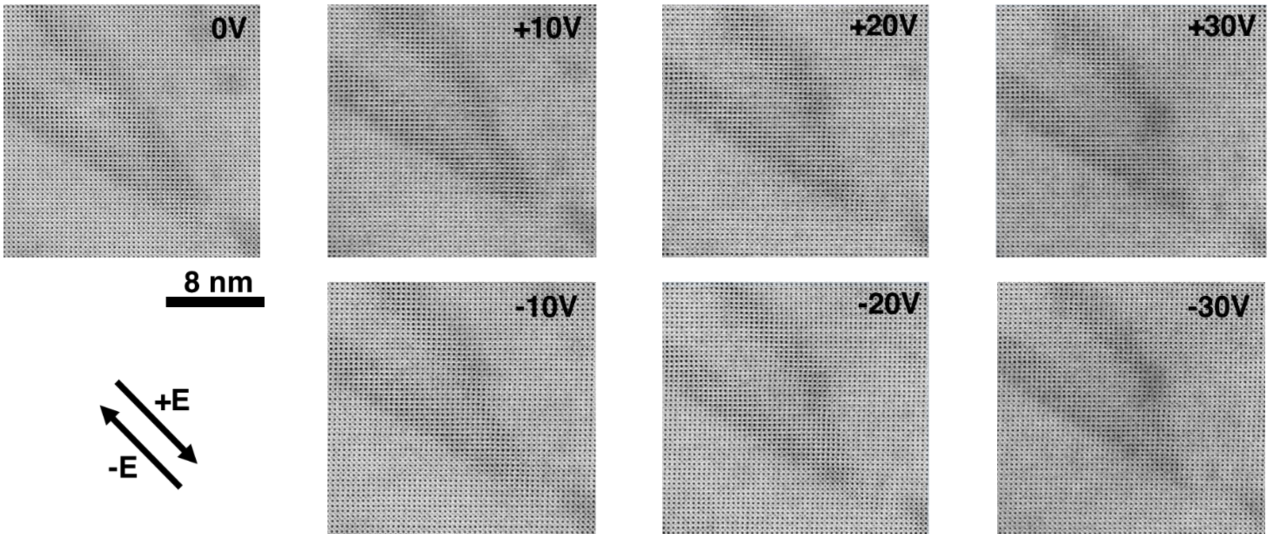

Figure S 7.1 Atomic resolution BF images at the wall location for 0V, +10V, -10V, +20V, -20V, +30V and -30V sequence. The BF images for 0V, +30V and -30V have been already shown in the main manuscript but for the sake of comparison we decided to show them together with the images for the other voltages.

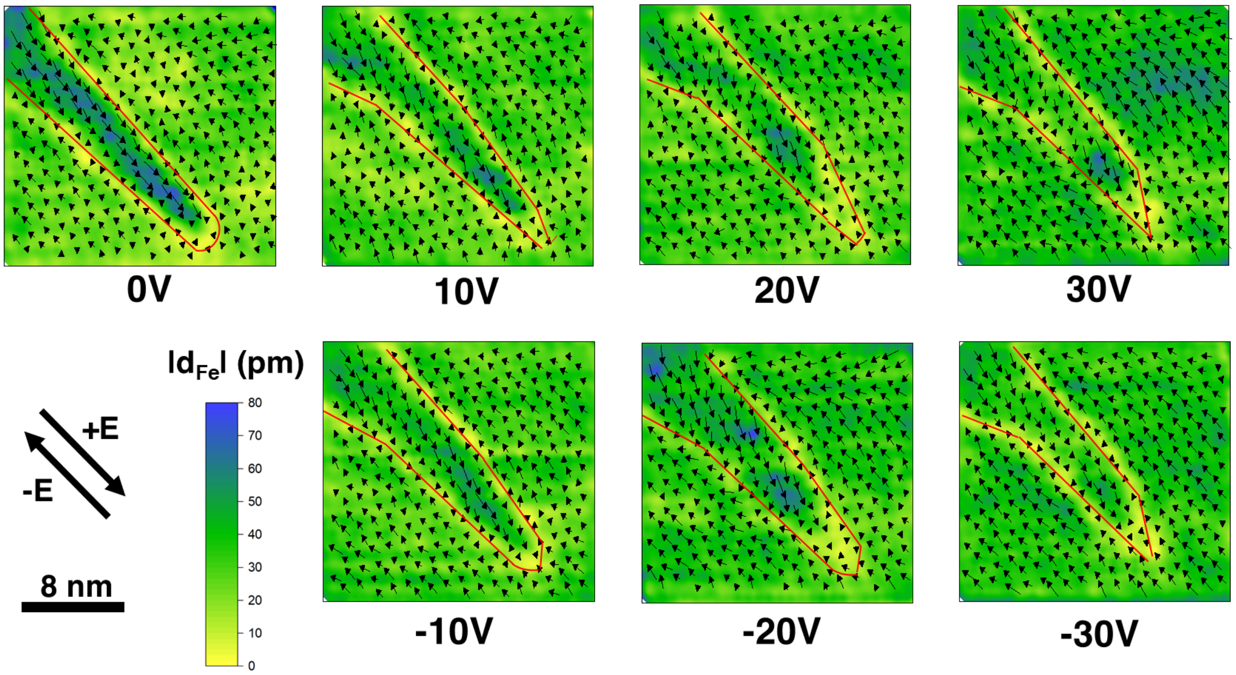

Figure S 7.2 Fe-displacement vector map (black arrows) overlaid on colored map of the Fe-displacement magnitude for 0V, 10V, -10V, 20V, -20V, 30V and -30V sequence. The DW region is marked with red line. The direction of the electric field is indicated by black arrows. The Fe-displacement vector map for 0V, 30V and -30V have been already shown in the main manuscript but for the sake of comparison we decided to show them together with the maps for the other voltages.

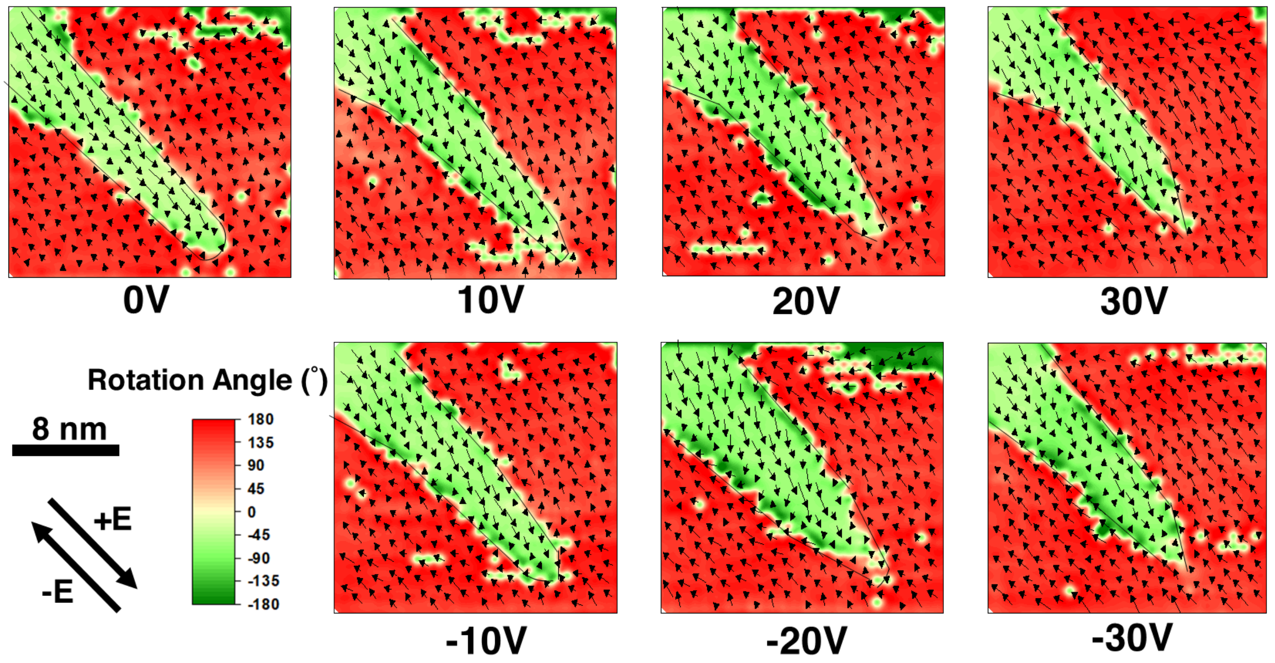

Figure S 7.3 Fe-displacement vector map (black arrows) overlaid on colored map of the Fe-displacement rotation angle for 0V, 10V, -10V, 20V, -20V, 30V and -30V sequence. The DW region is marked with black line. The direction of the electric field is indicated by black arrows.

Supplementary 8- **Estimations of the normal component of the Fe-displacement before and after the application of the electric field for the experiment following the tip of the zigzag DWs**

The amount of bound charge (σ) is given by the change of the normal polarization component to the DW plane: $-\sigma = \triangle \boldsymbol{P} \cdot \boldsymbol{n}$, where $\triangle \boldsymbol{P}$ is the difference between polarization of adjacent domains and $\boldsymbol{n}$ is the DW normal[13]. Polarization and Fe-displacement are in relation of direct proportionality[6], therefore, $-\sigma \sim \triangle \boldsymbol{d}$.

Three segments were considered to form the tip of the zigzag walls: DWI, DWII and apex. The normal component of Fe-displacement was calculated for each of these three segments at 0 and 30V.

The displacement vector (its magnitude and orientation) on either side of the wall was considered to be center of mass of the polar distribution (noted as $\boldsymbol{d}_{\boldsymbol{1},\boldsymbol{2}}$).

The plane of the wall was evaluated from Fe-displacement maps.

The normal Fe-displacement component is: $\Delta\, \boldsymbol{d} = \boldsymbol{d}_{\boldsymbol{1n}}^{\boldsymbol{I,II,apex}} - \boldsymbol{d}_{\boldsymbol{2n}}^{\boldsymbol{I,II,apex}}$, where $\boldsymbol{d}_{\boldsymbol{1n}}^{\boldsymbol{I,II,apex}}$ and $\boldsymbol{d}_{\boldsymbol{2n}}^{\boldsymbol{I,II,apex}}$ represents the normal component of the Fe-displacement of Domain 1 and Domain 2, respectively, relative to the plane of DWI, DWII or the apex.

$\left|\boldsymbol{d}_{\boldsymbol{1,2n}}^{\boldsymbol{I,II,apex}}\right|$is calculated as $\left|\boldsymbol{d}_{\boldsymbol{1,2n}}^{\boldsymbol{I,II,apex}}\right| = \left|\boldsymbol{d}_{\boldsymbol{1,2}}\right| \sin\theta_{1,2}^{I,II,apex}$ where $\theta_{1,2}^{I,II,apex}$ is the angle between the $\boldsymbol{d}_{\boldsymbol{1,2}}$ vectors and the wall plane DWI, DWII or apex.

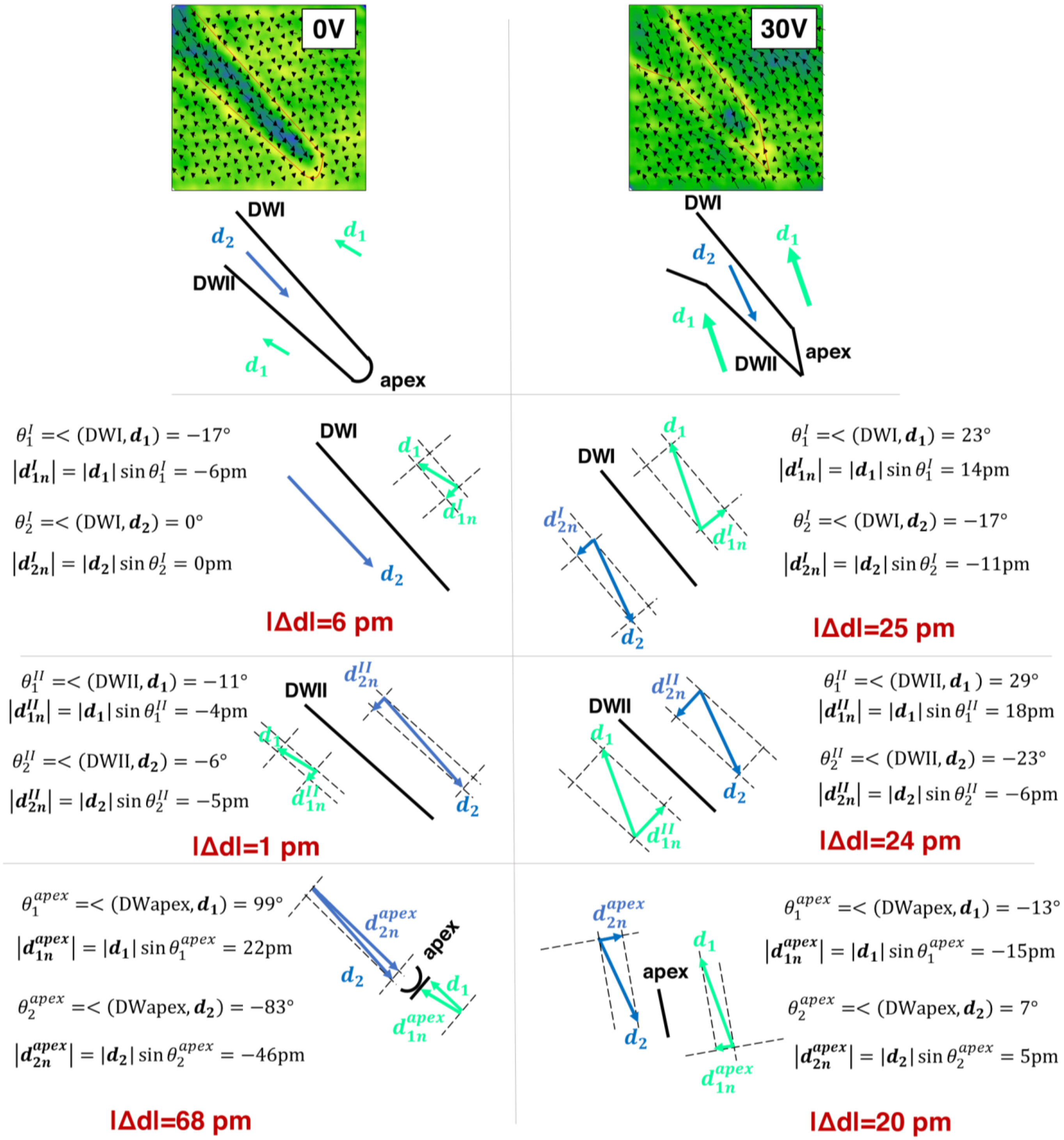

0V
30V
DWI
DWII
apex
d_1
d_2
$\theta_1^I = \angle(\mathrm{DWI}, \boldsymbol{d_1}) = -17°$
$|\boldsymbol{d_{1n}^I}| = |\boldsymbol{d_1}| \sin\theta_1^I = -6\mathrm{pm}$
$\theta_2^I = \angle(\mathrm{DWI}, \boldsymbol{d_2}) = 0°$
$|\boldsymbol{d_{2n}^I}| = |\boldsymbol{d_2}| \sin\theta_2^I = 0\mathrm{pm}$
|Δd|=6 pm
$\theta_1^I = \angle(\mathrm{DWI}, \boldsymbol{d_1}) = 23°$
$|\boldsymbol{d_{1n}^I}| = |\boldsymbol{d_1}| \sin\theta_1^I = 14\mathrm{pm}$
$\theta_2^I = \angle(\mathrm{DWI}, \boldsymbol{d_2}) = -17°$
$|\boldsymbol{d_{2n}^I}| = |\boldsymbol{d_2}| \sin\theta_2^I = -11\mathrm{pm}$
|Δd|=25 pm
$\theta_1^{II} = \angle(\mathrm{DWII}, \boldsymbol{d_1}) = -11°$
$|\boldsymbol{d_{1n}^{II}}| = |\boldsymbol{d_1}| \sin\theta_1^{II} = -4\mathrm{pm}$
$\theta_2^{II} = \angle(\mathrm{DWII}, \boldsymbol{d_2}) = -6°$
$|\boldsymbol{d_{2n}^{II}}| = |\boldsymbol{d_2}| \sin\theta_2^{II} = -5\mathrm{pm}$
|Δd|=1 pm
$\theta_1^{II} = \angle(\mathrm{DWII}, \boldsymbol{d_1}) = 29°$
$|\boldsymbol{d_{1n}^{II}}| = |\boldsymbol{d_1}| \sin\theta_1^{II} = 18\mathrm{pm}$
$\theta_2^{II} = \angle(\mathrm{DWII}, \boldsymbol{d_2}) = -23°$
$|\boldsymbol{d_{2n}^{II}}| = |\boldsymbol{d_2}| \sin\theta_2^{II} = -6\mathrm{pm}$
|Δd|=24 pm
$\theta_1^{apex} = \angle(\mathrm{DWapex}, \boldsymbol{d_1}) = 99°$
$|\boldsymbol{d_{1n}^{apex}}| = |\boldsymbol{d_1}| \sin\theta_1^{apex} = 22\mathrm{pm}$
$\theta_2^{apex} = \angle(\mathrm{DWapex}, \boldsymbol{d_2}) = -83°$
$|\boldsymbol{d_{2n}^{apex}}| = |\boldsymbol{d_2}| \sin\theta_2^{apex} = -46\mathrm{pm}$
|Δd|=68 pm
$\theta_1^{apex} = \angle(\mathrm{DWapex}, \boldsymbol{d_1}) = -13°$
$|\boldsymbol{d_{1n}^{apex}}| = |\boldsymbol{d_1}| \sin\theta_1^{apex} = -15\mathrm{pm}$
$\theta_2^{apex} = \angle(\mathrm{DWapex}, \boldsymbol{d_2}) = 7°$
$|\boldsymbol{d_{2n}^{apex}}| = |\boldsymbol{d_2}| \sin\theta_2^{apex} = 5\mathrm{pm}$
|Δd|=20 pm

(1) Moering, J.. NEW FIB-OPTIMIZED E-CHIPS, 2018 https://www.protochips.com/news/fib-optimized-e-chips/ (accessed 2022-07-19).
(2) Ignatans, R.; Damjanovic, D.; Tileli, V. Local Hard and Soft Pinning of 180° Domain Walls in $BaTiO_3$ Probed by *in Situ* Transmission Electron Microscopy. *Phys. Rev. Materials* **2020**, *4* (10), 104403.
(3) Vogel, A.; Sarott, M. F.; Campanini, M.; Trassin, M.; Rossell, M. D. Monitoring Electrical Biasing of $Pb(Zr_{0.2}Ti_{0.8})O_3$ Ferroelectric Thin Films In Situ by DPC-STEM Imaging. *Materials* **2021**, *14* (16), 4749.
(4) Li, L.; Xie, L.; Pan, X. Real-time studies of ferroelectric domain switching: a review. *Rep. Prog. Phys.* **2019**, *82*, 26502.
(5) Rojac, T.; Bencan, A.; Drazic, G.; Sakamoto, N.; Ursic, H.; Jancar, B.; Tavcar, G.; Makarovic, M.; Walker, J.; Malic, B.; Damjanovic, D. Domain-Wall Conduction in Ferroelectric $BiFeO_3$ Controlled by Accumulation of Charged Defects. *Nat. Mater.* **2017**, *16* (3), 322–327.
(6) Nelson, C. T.; Winchester, B.; Zhang, Y.; Kim, S.-J.; Melville, A.; Adamo, C.; Folkman, C. M.; Baek, S.-H.; Eom, C.-B.; Schlom, D. G.; Chen, L.-Q.; Pan, X. Spontaneous Vortex Nanodomain Arrays at Ferroelectric Heterointerfaces. *Nano Lett.* **2011**, *11* (2), 828–834.
(7) Catalan, G.; Scott, J. F. Physics and Applications of Bismuth Ferrite. *Adv. Mater.* **2009**, *21* (24), 2463–2485.
(8) Streiffer, S. K.; Parker, C. B.; Romanov, A. E.; Lefevre, M. J.; Zhao, L.; Speck, J. S.; Pompe, W.; Foster, C. M.; Bai, G. R. Domain Patterns in Epitaxial Rhombohedral Ferroelectric Films. I. Geometry and Experiments. *J. Appl. Phys.* 1998, *83* (5), 2742–2753.
(9) Taherinejad, M.; Vanderbilt, D.; Marton, P.; Stepkova, V.; Hlinka, J. Bloch-Type Domain Walls in Rhombohedral $BaTiO_3$. *Phys. Rev. B* **2012**, *86* (15), 155138.
(10) Wang, W.-Y.; Tang, Y.-L.; Zhu, Y.-L.; Xu, Y.-B.; Liu, Y.; Wang, Y.-J.; Jagadeesh, S.; Ma, X.-L. Atomic Level 1D Structural Modulations at the Negatively Charged Domain Walls in $BiFeO_3$ Films. *Adv. Mater. Interfaces* **2015**, *2* (9), 1500024.
(11) Condurache, O.; Dražić, G.; Sakamoto, N.; Rojac, T.; Benčan, A. Atomically Resolved Structure of Step-like Uncharged and Charged Domain Walls in Polycrystalline $BiFeO_3$. *J. Appl. Phys.* **2021**, *129* (5), 054102.
(12) Jia, C.-L.; Jin, L.; Wang, D.; Mi, S.-B.; Alexe, M.; Hesse, D.; Reichlova, H.; Marti, X.; Bellaiche, L.; Urban, K. W. Nanodomains and Nanometer-Scale Disorder in Multiferroic Bismuth Ferrite Single Crystals. *Acta Mater.* **2015**, *82*, 356–368.
(13) *Topological Structures in Ferroic Materials*; Seidel, J., Ed.; Springer Series in Materials Science; Springer International Publishing: Cham, **2016**; Vol. 228.
